# Supplementary material for: A COVID-19 Outbreak in a Large Meat-Processing Plant in England: Transmission Risk Factors and Controls
Source: Int J Environ Res Public Health. 2023 Sep 22;20(19):6806. doi: 10.3390/ijerph20196806 (PMC10572747; doi:10.3390/ijerph20196806)
Supplement: Supplementary file 1 [file ijerph-20-06806-s001.zip › ijerph-2491346-supplementary.pdf]

SUPPLEMENTARY MATERIAL

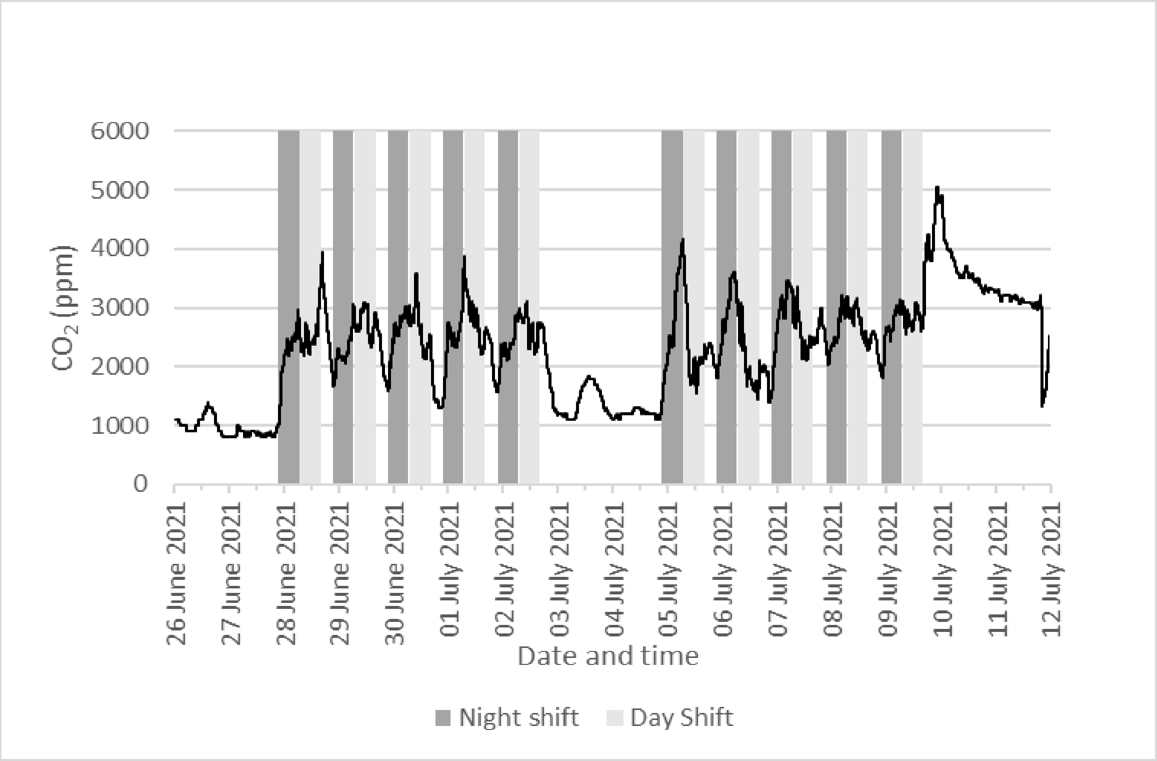

Figure S1: Real-time CO_2 measurements in the cut-preparation area of the meat processing plant between 25 June 2021 and 11 July 2021. The line graph shows 30-minute rolling average of CO_2 concentrations. The dark grey area covers night-shift time (Sunday – Friday, 21:30-06:30), the light grey area covers day-shift time (Monday – Friday, 07:00-16:00), and the white area is no working shift. This area was separated by flexible plastic drapes from the main production area (A) where CO_2 was used in the packaging process. Due to the potential influence of the exogenous CO_2 sources from the adjacent main production area, the CO_2 measurements in this area could not be used to assess the adequacy of ventilation. At some point, the instrument used to measure CO_2 was contaminated with chicken debris and might affect the reading after 09 July, which did not decrease significantly at the weekend. Therefore, the peak reading of 5100ppm on 10 July could be a measurement error.

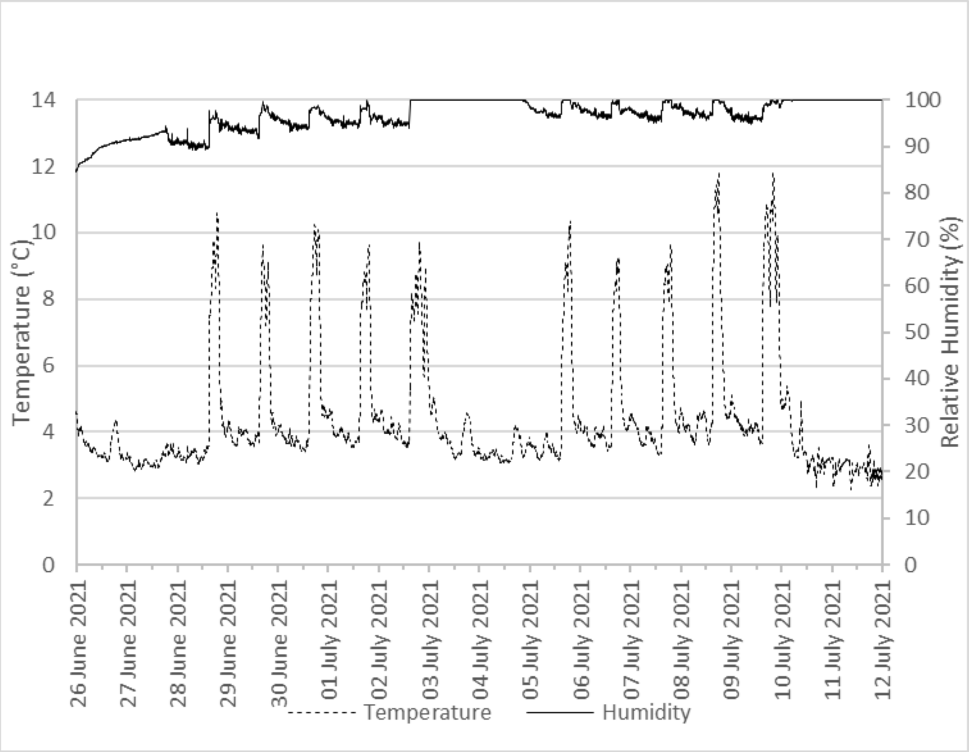

Figure S2: Real-time measurements of temperature and relative humidity (RH) in the cut-preparation area of the meat processing plant between 25 June 2021 and 11 July 2021.The top line graph shows RH with a mean of 95.9% (range: 67.5-100%). The lower line graph shows the room temperature changes with a mean 4.5°C (range: 2.3-11.9°C).

Table S1: Measurement results of the workplace environmental conditions in the meat processing plant following a COVID-19 outbreak. Spot measurements were conducted between 9.30 and 12.00 on 25 June 2021, and real-time measurements were carried out between 25 June and 11 July 2021.

**Site area**	**Spot measurements**
	**Noise level (dB(A))**
Meat material intake	80
Main production area A, in-between conveyors	81
Packing area	83
	**CO_2 level (ppm)**
Main production area A by packing line machines [a]	2800
Meat material intake [a]	2500
Raised central area of production area B [a]	2500
Cut-preparation area by cutting bench beneath conveyor [b]	2300
Main corridor towards production areas and main washing areas [b]	1900
Washing area by the sinks between office area and production entrance [b]	1500
Lobby by the hand wash at the entrance to the production areas [b]	1200
Production exit to the lobby by the entrance to the changing areas	1000
Main locker	1000
Engineering workshop	800

**Site area**	**Real-time measurements, mean & range**		
	**CO_2 level (ppm)**	**Temperature (°C)**	**Humidity (%RH)**
Cut-preparation area by cutting bench beneath conveyor [b]	2268 (400-4100)	4.5 (2.3-11.9)	95.9 (67.5-100)
Main washing area for production staff [b]	605 (300-1800)	16.7 (13.7-19.4)	69.1 (55.0-83.2)
Washing area above sinks at the office-production entrance [b]	1126 (400-2500)	10.9 (8.9-13.4)	59.4 (49.6-77.9)
Canteen, on top of vending machines	388 (300-800)	20.7 (16.7-27.6)	65.1 (33.6-81.9)
Main locker room near main entrance area	516 (300-1400)	18.6 (16.1-27.6)	Error reading

[a] These were the main production areas where CO_2 was used in the packaging process. [b] These were areas connected to the main production areas. The CO_2 readings from both a and b areas cannot be used to assess the adequacy of ventilation.
